# A Simple Test of Class-Level Genetic Association Can Reveal Novel Cardiometabolic Trait Loci

**DOI:** 10.1371/journal.pone.0148218

**Published:** 2016-02-09

**Authors:** Jing Qian, Sara Nunez, Eric Reed, Muredach P. Reilly, Andrea S. Foulkes

**Affiliations:** 1 Department of Biostatistics and Epidemiology, University of Massachusetts, Amherst, MA, United States of America; 2 Department of Mathematics and Statistics, Mount Holyoke College, South Hadley, MA, United States of America; 3 Department of Medicine, Columbia University, New York, NY, United States of America; National Taiwan University, TAIWAN

## Abstract

**Background:**

Characterizing the genetic determinants of complex diseases can be further augmented by incorporating knowledge of underlying structure or classifications of the genome, such as newly developed mappings of protein-coding genes, epigenetic marks, enhancer elements and non-coding RNAs.

**Methods:**

We apply a simple class-level testing framework, termed Genetic Class Association Testing (GenCAT), to identify protein-coding gene association with 14 cardiometabolic (CMD) related traits across 6 publicly available genome wide association (GWA) meta-analysis data resources. GenCAT uses SNP-level meta-analysis test statistics across all SNPs within a class of elements, as well as the size of the class and its unique correlation structure, to determine if the class is statistically meaningful. The novelty of findings is evaluated through investigation of regional signals. A subset of findings are validated using recently updated, larger meta-analysis resources. A simulation study is presented to characterize overall performance with respect to power, control of family-wise error and computational efficiency. All analysis is performed using the GenCAT package, R version 3.2.1.

**Results:**

We demonstrate that class-level testing complements the common first stage minP approach that involves individual SNP-level testing followed by post-hoc ascribing of statistically significant SNPs to genes and loci. GenCAT suggests 54 protein-coding genes at 41 distinct loci for the 13 CMD traits investigated in the discovery analysis, that are beyond the discoveries of minP alone. An additional application to biological pathways demonstrates flexibility in defining genetic classes.

**Conclusions:**

We conclude that it would be prudent to include class-level testing as standard practice in GWA analysis. GenCAT, for example, can be used as a simple, complementary and efficient strategy for class-level testing that leverages existing data resources, requires only summary level data in the form of test statistics, and adds significant value with respect to its potential for identifying multiple novel and clinically relevant trait associations.

## Introduction

Large-scale genome-wide association (GWA) meta-analyses have become routine practice for discovery of the genetic underpinnings of complex traits, such as cardiometabolic disease (CMD). Several resulting meta-analysis resources, including summary level information on association between each of several million typed and imputed SNPs and a well-defined trait, are now publicly available. At the same time, we see a growing number of classifications or taxonomies of the genome—for example, protein coding genes, epigenetic marks, enhancer elements and non-coding RNAs—herein referred to as *classes*. Additionally, we have refined knowledge regarding the linkage-disequilibrium (LD) structure across the genome via the 1000 genomes project. In turn, this presents new opportunity to further potentiate the extensive, existing data resources through application of theoretically sound methodological advancements that integrate these multiple knowledge components. In this report, we leverage these multiple existing knowledge resources to demonstrate the potential added value of applying a class-level testing strategy to complement more routine analysis practice.

**Fig 1:**
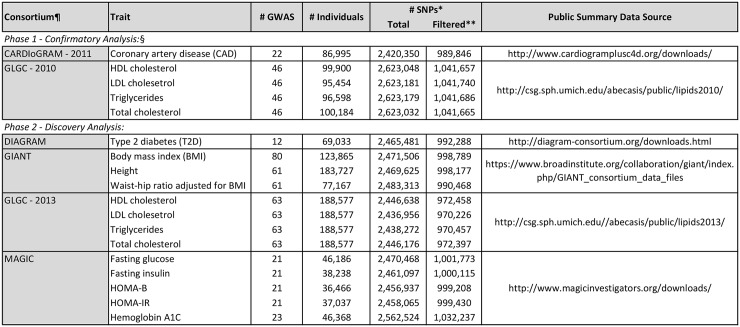
Summary of GWAS meta-analysis data resources. ¶Acronyms: The Coronary ARtery DIsease Genome-wide Replication And Meta-analysis (CARDIoGRAM) consortium data; DIAbetes Genetics Replication And Meta-analysis (DIAGRAM) consortium data; The Genetic Investigation of ANthropometric Traits (GIANT) consortium meta-analysis data; The Global Lipids Gentics Consortium (GLGC) meta-analysis data; and Meta-Analyses of Glucose and Insulin-related traits Consortium (MAGIC) meta-analysis data. §The CARDioGRAM—2011 and GLGC—2010 summary data are used in the *phase one—discovery analysis* as expanded resources based on larger numbers of individuals are now available to validate GenCAT “discoveries” using these older data resources. The remaining data sets are used in the *phase two—discovery analysis* which yields the reported novel findings. *Total number of typed and imputed SNPs in original data files available for download. **SNPs after filtering for HWE, MAF, call rate, belonging to a protein-coding gene and in PennCATH HapMAP imputed data.

To illustrate the breadth of potential novel discoveries with class-level testing, we test for protein-coding gene associations with 14 unique phenotypes across six publicly-available meta-analysis summary level data resources focused on the genetic basis of complex CMD, summarized in Fig 1. A *confirmatory analysis* is presented that leverages the multiple distinct analysis phases involving expanded cohorts that are reported for both the Global Lipids Gentics Consortium (GLGC) meta-analysis data [1, 2] and The Coronary ARtery DIsease Genome-wide Replication And Meta-analysis (CARDIoGRAM) consortium data [3, 4], as described in more detail in the methods section below. A *discovery analysis* uses the recently expanded GLGC data (GLGC—2013) [2], the DIAbetes Genetics Replication And Meta-analysis (DIAGRAMv3) consortium data [5, 6], the Genetic Investigation of ANthropometric Traits (GIANT) consortium meta-analysis data [7–9] and Meta-Analyses of Glucose and Insulin-related traits Consortium (MAGIC) meta-analysis data [10, 11]. Protein coding gene-level associations with each of the 14 traits listed in Fig 1 are investigated. These public resources represent the largest sets of genome wide data for traits and diseases that collectively are the greatest source of morbidity and mortality worldwide. Using the single-element analysis procedures, GWA studies have identified many novel loci for these traits, all with complex genetic bases. Despite these large resources and substantial discoveries, the majority of the heritability for several of these traits remains unexplained, highlighting the need for additional studies and application of statistical methods to reveal more completely the genetic architecture of these disease-related traits.

The testing framework we apply, termed Genetic Class Association Testing (GenCAT), leverages the available meta-analysis results for each of the resources listed in Fig 1. These findings include individual SNP-level test statistics of association based on combined output from fitting generalized linear multivariable models for each SNP, adjusting for clinical and demographic information, in each of multiple data sets (numbers provided in Fig 1). GenCAT is a simple extension of the previously described quadratic test (QT) and the versatile gene-based association study (VEGAS) approach [12, 13]. Similar to the QT, GenCAT involves first transforming normal variates using estimates of underlying within class correlation structures. The QT approach is based on inverse normally transformed *p*-values from SNP level tests of the difference in allele frequencies between cases and controls, while we use test statistics from generalized linear models fitted to binary and quantitative traits. This requires an alternative formation of the associated covariance structure, as derived in Appendix A. Additionally we introduce a data reduction component that minimizes data redundancies, and thus instabilities in the transformations, introduced by high degrees of within class correlations. This approach incorporates the eigenvalues of the covariance matrix, a measure also used in [14] and [15], but is principally different than these approaches, as discussed below. This data transformation allows straightforward application of a theoretically derived test statistic distribution for formal hypothesis testing of individual class-level effects, rendering the approach computationally efficient and thus markedly distinct from VEGAS which relies on an empirically based testing strategy which is computationally more intensive, as illustrated in the simulation section below.

A growing literature exists on gene and pathway-based approaches to GWA analysis (see for example, [12, 13, 16–29]), each with unique attributes and notable, relative advantages and disadvantages. These include methods specifically developed for rare-variant analysis (which is not a focus of this manuscript), and methods that include use of raw genotype data as well as those that leverage summary level data resources, as we do herein. The purpose of this manuscript is not to provide a comprehensive comparison of these methods, discerning which is most powerful under given alternatives; rather, we aim to (1) highlight the potential gains from application of one gene-based strategy, namely GenCAT, when coupled with the common first-stage analysis that focuses on single SNP associations, termed minP; and (2) report on a comprehensive application of this gene-based strategy to several large meta-analysis resources involving CMD related traits. The minP approach to evaluating association between protein coding genes and a trait is to ascribe SNPs with p-values less than a Bonferroni corrected threshold to genes and to declare these genes statistically meaningful. Typically a genome-wide significant threshold is set to be 5×10^−8^, which assumes roughly 1-million independent signals across the genome. While the minP approach has led to a large number of novel gene discoveries, it is limited in that a gene must contain at least one SNP with a very large signal to be identified, and may not capture genes with multiple SNPs that have moderate signals, which in combination are genetically, biologically, clinically, and statistically meaningful. We focus on GenCAT as our testing strategy in particular because it is easy to implement with existing data resources, computationally efficient and powerful under alternatives not well captured by minP, as we illustrate in our simulation study, and extensible—e.g. we illustrate the flexibility and efficiency of applying GenCAT in a pathway analysis. However, we acknowledge that alternative methods may perform equally well or better under certain alternatives. We consider the comparative computational efficiency of VEGAS [13] and the family-wise error control of QT [12] in our simulation study. The overarching emphasis herein is on the value of implementing a gene-level strategy as routine best practice for GWA analysis.

## Methods

### A class-level test statistic

Consider a class of size *n* and let **z** = (*z*_1_, *z*_2_, …, *z*_*n*_)^*T*^ be a vector of *n* test statistics (z-scores) for association of each element in this class with the trait under study. For simplicity of notation we suppress dependency on class. Typically, the elements of **z** are SNP-level Wald test statistics arising from fitting multivariable models, where each model includes a single SNP term, as well as several clinical and demographic variables. The vector **z** has a multivariate normal distribution, **z** ~ *N*_n_(0, Ʃ), under the null of no association between any of the *n* elements in this class. This assumption of normality is reasonable given the large sample sizes of the GWA meta-anlayses (see Fig 1). Because Σ is square and positive definite, we can decompose Σ as follows using the eigenvalue decomposition, $\Sigma =Q\Lambda Q^{T}$, where *Q* is an orthogonal matrix (i.e., $Q^{T}Q=QQ^{T}=I$) whose columns are the eigenvectors (normalized to unit length) of Σ, and Λ is a diagonal matrix with diagonal elements equal to the eigenvalues of Σ. We define the class-level test statistic, $C_{s}$, as the sum of the squared transformed test statistics, $C_{s}=U^{T}U$, where $U=\Lambda^{-1/2}Q^{T}z$. We note that $C_{s}$ can be expressed as $C_{s}=z^{T}{\left(\Lambda^{-1/2}Q^{T}\right)}^{T}\left(\Lambda^{-1/2}Q^{T}\right)z=z^{T}\Sigma^{-1}z$ and the quadratic form, $z^{T}\Sigma^{-1}z$, follows a central chi-squared distribution with *n* degrees of freedom, i.e., *χ*^2^(*n*).

To calculate $C_{s}$, we replace the variance-covariance matrix of *z*-test statistics, Σ, with *P* where the (*r*, *s*)-th component of *P* is Pearson’s correlation coefficient, *ρ*_*x*_*r*_, *x*_*s*__ between SNPs *r* and *s*. The related linkage disequilibrium (LD) structure based on phase known data, has been applied previously without formal proof, as an estimate of the positive correlation between SNP-level *χ*^2^-test statistics in genes (for example, [13, 15]). In our setting, we are interested in positive and negative correlation of SNP-level *z*-test statistics in genes, in the context of multivariable modeling of additive genetic effects. In Appendix A, we provide a detailed derivation for the equivalence under certain conditions of the pairwise correlation between the *z*-test statistics and the pairwise correlation between the SNP measures in the linear model framework. While this derivation is more complex in the generalized linear model (GLM) setting, we completed a simulation study to show that the expected correlation between test statistics based on a GLM with a logit link function is estimated by the observed SNP-level correlation. This results is illustrated in S1 Fig, with simulation details provided in the corresponding figure legend. Notably, use of pairwise SNP correlations to estimate the correlations of corresponding test statistics in the GLM setting has been described and applied, (for example, [16]).

In the example herein, we are analyzing summary level data, and therefore Σ needs to be estimated using an independent data set with raw genotype information. Selecting an appropriate dataset with similar ancestry for estimation of Σ is imperative as use of a biased estimate of Σ based on data derived from individuals with different racial or ethnic backgrounds could lead to erroneous conclusions. In our example we use the PennCATH data for estimation of Σ. PennCath is one of the core GWAS nested in CARDIoGRAM and serves as a representative regional population with no admixture [3, 30]. We use $\hat{P}$ to represent this estimate of Σ in which pairwise correlations are estimated from a representative sample.

When Σ is close to singular resulting from a very high degree of correlation between at least two test statistics, the transformed values *U*, and therefore the test statistic $C_{s}$ can be unstable. We eliminate redundancies arising from very high degrees of within class correlations using a dimension reduction approach that relies on the eigenvalues of the covariance matrix. This measure is also used in [14] and [15]; however, our approach is principally different because rather than determining the effective number of tests using just these eigenvalues, we are mapping our full data onto a reduced dimensional space, defined by the eigenvectors that captures a pre-specified proportion of the within class variability. Specifically, we project the vector of test statistics onto the space defined by the minimum set of eigenvectors of Σ that capture (1−*ψ*)% of the variability in Σ, where in our example we let *ψ* = 0.05. That is, let $\lambda_{\left(1\right)},\cdots ,\lambda_{\left(n\right)}$ represent the ordered eigenvalues of Σ (from largest to smallest) and $q_{\left(1\right)},\cdots ,q_{\left(n\right)}$ the corresponding eigenvectors normalized to unit length. We select *K* such that:

K=mink∈[1,n]:∑i=k+1nλ(i)∑i=1nλ(i)<ψ (1)

We define $\tilde{\Lambda }$ to be the sub-matrix of Λ with diagonal elements $\lambda_{\left(1\right)},\cdots ,\lambda_{\left(K\right)}$, and let $\tilde{Q}$ be the sub-matrix of *Q* with columns $q_{\left(1\right)},\cdots ,q_{\left(K\right)}$. Finally, we let:

$$\begin{array}{c} \tilde{U}={\tilde{\Lambda }}^{-1/2}{\tilde{Q}}^{T}z \end{array}$$ (2)

and use this in place of *U* for our calculations of $C_{s}$ where ${\tilde{U}}_{i}$ is the *i*th element of $\tilde{U}$. This data reduction procedure serves ultimately to improve computational efficiency as well as stability of our test statistics. A step-by-step summary of GenCAT is provided in Box 1.

**Box 1: Summary of GenCAT approach (for each class).**

1. Calculate $\tilde{U}$ of Eq (2) as follows:
  - Compute the eigenvalues and eigenvectors of Σ. Let $\lambda_{\left(1\right)},\cdots ,\lambda_{\left(n\right)}$ represent the eigenvalues sorted from largest to smallest.
  - Determine the minimum value of *K* such that the sum of *n*−*K* smallest eigenvalues is less than *ψ* = 5% of the sum of all eigenvalues (Eq (1))
  - Define the reduced matrices $\tilde{\Lambda }$ and $\tilde{Q}$ to include the largest *K* eigenvalues and corresponding eigenvectors given in Λ and *Q*.
2. Calculate the class-level test statistic, *C*_*s*_ defined as the sum of squared transformed test statistics:
  $$\begin{array}{c} {\tilde{C}}_{s}={\tilde{U}}^{T}\tilde{U} \end{array}$$
  and compare to a central chi-squared distribution with *K* degrees of freedom to determine an associated p-value.
3. Compare the p-value of step 2 to a Bonferroni adjusted threshold based on the total number of classes.

### Analysis approach

We begin with a *phase 1—confirmatory analysis* that aims to leverage the multiple phases of data collection and resulting meta-analysis data resources and publications that are available through both the GLGC and CARDIoGRAM consortia. This first stage validation analysis involves applying GenCAT to early phase analysis results, specifically the 2010 GLGC findings reported in [1] and the 2011 CARDIoGRAM findings reported in [3] (GLGC—2010 and CARDIoGRAM—2011 in Fig 1), and then using expanded resources that are based on larger and more recently reported cohort data [2–4] as a comparator for evaluating the performance of GenCAT. While GenCAT is intended as a complementary strategy to minP, we expect true GenCAT positive findings to be supported by minP positive results in larger data settings and thus we consider this comparison. For GLGC, the expanded data resources used for validation are available for direct interrogation (GLGC—2013 in Fig 1), while for CARDIoGRAM we rely on published reports including an expanded replication study involving up to 56,682 additional individuals, reported in [3], and a larger cohort study of 63,746 CAD cases and 130,681 controls representing an expansion of the CARDIoGRAM—2011 data to include 34 additional studies, reported in [4]. For both the GLGC—2010 and the original CARDIoGRAM—2011 studies, we report the numbers of novel GenCAT discoveries that are subsequently discovered using minP in the expanded data resources involving substantially larger sample sizes. The confirmatory analysis is intended to characterize the performance of GenCAT for identifying protein-coding gene associations with CMD traits that are later discoverable (after additional data collection) using standard analysis tools.

In a *phase 2—discovery analysis*, we apply GenCAT to 13 CMD related traits across four GWA meta-analysis resources (described in Fig 1). We report the total number of protein-coding genes that are GenCAT+, i.e. the number of gene-level test statistics, ${\tilde{C}}_{s}∼\chi^{2}\left(K\right)$, with a corresponding p-value less than 0.05/*N*, where *N* is the number of genes investigated for the corresponding GWA study. Additionally, the numbers of these GenCAT discoveries that are minP-, i.e. do not contain a single SNP p-value less than 5×10^−8^, are reported for each trait. GenCAT+ genes in novel loci are defined as a subset of the GenCAT+/minP- genes that are also not within a ±500Kb region of a minP+ gene. Finally, GenCAT+ novel gene findings within a ±500Kb region are combined into novel loci and the total number of novel loci are reported. We also provide detailed results, including gene names, coordinates, numbers of SNPs, GenCAT statistic and p-value, and minimum single SNP p-value, for GenCAT+ genes within GenCAT novel loci. We acknowledge that our discovery analysis does not interrogate intergenic regions which are increasingly being annotated and recognized as containing highly ordered regulatory elements that control expression and function of protein-coding genes and in themselves can be actively transcribed molecules. We consider this further in the Discussion below.

The input to our analysis is single SNP-level test statistics in the form of z-scores corresponding to tests of additive association between single SNPs and a specified trait. These statistics are previously derived from fitting generalized linear multivariable models in each of multiple sub-studies and combing these in a meta-analysis. The CARDIoGRAM data, for example, are the result of a meta-analysis of 86,995 individuals (22,233 cases and 64,762 controls) across 22 GWA studies that tested trait association at a total of 2,420,350 genotyped and imputed SNPs that span both protein coding genes as well as intergenic regions. We focus our analysis on 989,932 SNPs that are located in 17,280 protein coding genes and present in the PennCath cohort data [30]. PennCath is one of the 22 studies that is included in the CARDIoGRAM GWA meta-analysis and we have direct access to delinked raw genotype data to allow estimation of pairwise correlations between SNP level test statistics in this study as required to derive the GenCAT test statistic. Thus the PennCATH data are used for estimating correlations for each of the five meta-analysis resources. In addition to filtering SNPs in protein-coding genes, we limit analysis to SNPs that, in the PennCath cohort, have a minor allele frequency >0.01, a Hardy Weinberg equilibrium p-value <0.001, and a SNP call rate, defined as the proportion of non-missing values, ≥0.90. The total numbers of SNPs available, as well as the numbers after filtering, for each trait under study are provided in Fig 1.

### A simulation study

We conduct a simulation study to characterize the performance of GenCAT with respect to the family-wise error rate and power under a range of underlying conditions, with particular attention given to the relative performance of the commonly applied minP approach. Emphasis is on the added value of GenCAT and not a comparison of the two approaches as gene-level testing is intended to complement single-SNP analysis. Here the minP approach is defined in a standard way as calling a gene statistically significant if the minimum single-SNP p-value in the gene is less than a Bonferroni corrected threshold based on the total number of SNPs in the study. In practice the minP analysis typically proceeds by analyzing each SNP individually, and then ascribing genes *post hoc* to those SNPs that are statistically significant. We also highlight the potential gains in computational efficiency compared to the Versatile Gene-Based Association Study (VEGAS) approach, described by [13], which relies on simulation of the empirical distribution for p-value calculations.

The simulation study is based a complete set of 17,280 genes and the observed number of SNPs in the CARDIoGRAM consortium GWA meta-analysis data [3] of transformed test statistics within these genes. Raw genotype data from the PennCath cohort [30] are used to estimate the pairwise correlation between SNP-level test statistics as we have shown to be appropriate in Appendix A. Here we limit consideration to genes with less than 100 SNPs for computational purposes as we are repeating the simulation a large number of times; however, GenCAT can accommodate classes with a large number of components. To begin, for each gene *i*, we generate *k*_*i*_ independent z-scores from a *MVN*(0, *I*) distribution, where *k*_*i*_ is the number of SNPs in gene *i* after transformation using the PennCATH data to estimate the covariance structure. We assume multivariate normality to emulate the real data setting in which we have correlated, normally distributed test statistics. We calculate the GenCAT statistics ${\tilde{C}}_{s}$ and corresponding p-values and compare to the Bonferroni corrected threshold with an adjustment based on 17,280 genes. This process is repeated 4000 times and we determine the proportion of simulations that result in at least one significant gene finding (p-value <0.05/17280). These proportions serve as our estimates of the family-wise error rates under the complete null (FWEC).

Power in our setting is defined as the probability of detecting a single gene generated under the alternative. To determine empirical power, we first randomly select a gene *i* and generate *n*_*i*_ independent normals. Here we assume a pre-specified percentage, referred to as the “percentage partial signal”, arise from a $MVN(\mu 1_{n_{i}},I_{n_{i}})$ and the remaining arise from a $MVN(0,I_{n_{i}})$, where *n*_*i*_ is the number of observed SNPs in gene *i*. Calling these statistics $x_{i}^{1}$ and $x_{i}^{2}$ and using the estimated correlation structure, ${\hat{P}}_{i}$ from the PennCATH data, we then transform $x_{i}={\left(x_{i}^{1},x_{i}^{2}\right)}^{T}$ by pre-multiplying by $Q_{i}\Lambda_{i}^{1/2}$, where ${\hat{P}}_{i}=Q_{i}\Lambda Q_{i}^{T}$, *Q*_*i*_ is an orthogonal matrix whose columns are the eigenvectors (normalized to unit length) of ${\hat{P}}_{i}$, and Λ_*i*_ is a diagonal matrix with diagonal elements equal to the eigenvalues of ${\hat{P}}_{i}$. Formally, we have ${\tilde{x}}_{i}=Q_{i}\Lambda_{i}^{1/2}x_{i}$ and ${\tilde{x}}_{i}∼MVN\left({\tilde{\mu }}_{i},{\hat{P}}_{i}\right)$. These are treated as the observed data to which we apply GenCAT and record whether the gene is correctly selected, based on the Bonferroni corrected threshold of 0.05/17,280. Power is calculated assuming full signal for a range of *μ* from 2 to 6, and under partial signals of 0.1 to 0.9 for *μ* = 4.0. For each value of *μ*, and percentage partial signal, we conduct 2000 simulations, and power is defined as the proportion of the 2000 simulations that correctly detect association. Power is reported for ${\tilde{C}}_{s}$ as well as minP under the same conditions to characterize the potential contribution of a gene level testing strategy.

Finally, we determine the computational time for running VEGAS and GenCAT. Recall, VEGAS similarly defines a gene-level test statistic based on the sum of untransformed *χ*^2^ statistics in each gene; however, to determine statistical significance of each gene, the VEGAS approach requires the generation of new data—between 10^3^ and 10^7^ simulations—with the same correlation structure as the observed data and under the null model. The number of simulations for each depends on: (a) the total number of genes under study and (b) the level of significance of the specific gene being tested. So for example, a gene that is not significant (*p* > 0.10) will require 10^3^ simulations regardless of the total number of genes under study. On the other hand, a gene that has a p-value <10^−7^, will require 10^3^ simulations if it is the only gene being tested, but will require at least 10^7^ simulations if more than 10,000 genes are being considered to achieve appropriate precision.

To illustrate and contrast the computational time of each approach, we begin by considering the analysis time for each of two genes, Lipoprotein, Lp(A) (LPA) and Proline/Serine-Rich Coiled-Coil 1 (PSRC1) with 67 and 5 SNPs respectively, both highly significant based on minP and the $C_{s}$ statistic (results not shown) in CARDIoGRAM. For this study, we vary the assumed total number of genes under study from one to 10,000 and report the central processing unit (CPU)-time for analyzing just the single gene, using R on a single Intel Core i7-3520M CPU @ 2.90GHz. We also report the expected CPU-time for analysis of a complete set of 19,018 genes for an assumed range of between approximately 0.1% and 2.0% of genes with a p-value that is less than the Bonferroni corrected threshold.

### Available software

Analysis is performed using the GenCAT() function of the GenCAT package ver 1.0.1 in R (http://cran.r-project.org/web/packages/GenCAT/index.html). This function requires a data table with a row for each each SNP and columns corresponding to SNP name (e.g. rs number), SNP-level test statistic, chromosome number, class assignment, effect allele and other allele. Raw genotype data in the form of a SnpMatrix object is also required for estimation of the covariance. The resulting object includes several data frames, containing information on GenCAT test results, the SNPs used in analysis and transformed test statistics. The Manhattan plot is generated using the GenCAT_manhattan() function, also available in the GenCAT package.

## Results

### Applications to CMD traits in GWA meta-analysis resources

#### Findings of the *phase 1—confirmatory analysis*

**Fig 2:**
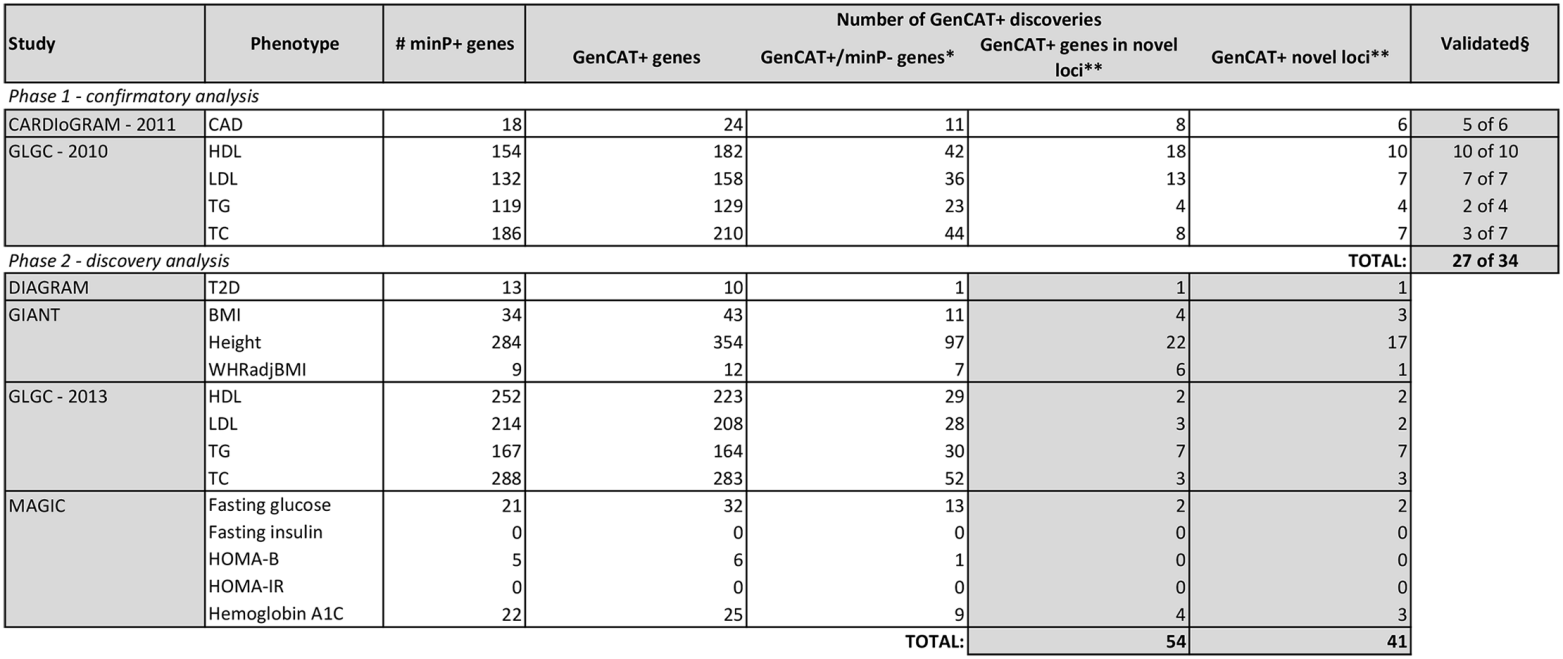
Summary of GenCAT discoveries in GWA meta-analysis data resources. *Number of genes detected by GenCAT that do not include a single SNP with a p-value less than the Bonferroni threshold of 5×10^−8^. **Number of genes/loci detected by GenCAT that are not within 500Kb of a gene that contains a single SNP with a p-value less than the Bonferroni threshold using the available GWA data. § Twenty-seven of 34 loci detected by GenCAT in CARDIoGRAM-2011 and GLGC-2010 were reported in follow-up analysis using minP with substantially more data [2–4]. These are therefore not novel findings given later publications, but highlight the ability of GenCAT to identify genes that are ultimately discoverable (“validated”) with minP when substantially larger datasets are interrogated.

A summary of the *phase 1—confirmatory analysis* findings is provided in the top half of Fig 2. In total, GenCAT identifies six novel loci for CAD using the available CARDIoGRAM meta-analysis summary data (see S1 Table for details). Five of these loci—labelled, ABO, MYL2, Collagen, Type IV, Alpha 1 (COL4A1), HHIP-Like 1 (HHIPL1) and ADAMTS7—are confirmed findings based on minP using expanded data resources [3, 4], while one of the GenCAT significant loci—Dual Specificity Phosphatase 26 (DUSP26)—is not reported as significant in follow-up analysis. Further investigation and validation of DUSP26 is required to confirm whether or not this represents a false finding. GenCAT analysis of the GLGC—2010 data suggests 28 novel loci, of which 23 are confirmed as minP+ using the expanded GLGC—2013 data resource (see S1 Table for details). Overall, these findings are consistent with our power calculations suggesting that GenCAT can detect associated loci with less data than minP in some circumstances. Or stated differently, here GenCAT finds trait associations with loci in smaller datasets and these associations are subsequently confirmed using the minP approach in much larger GWA datasets for the same traits.

#### Findings of the *phase 2—discovery analysis*

A summary of GenCAT findings across the 13 traits in the *phase 2—discovery analysis* are reported in the bottom half of Fig 2, with specific details provided in S2–S5 Tables. As GenCAT is intended to complement minP, we focus on the number of genes that were not detected by minP and are also not within 500Kb of a gene that is detected by minP. In total, GenCAT identifies 54 such genes, and these are mapped to 41 distinct loci (i.e., 41 regions that are not within 500Kb of one another.) Consistent with our expectation, the most discoveries are made in the large GIANT dataset for height, which is a highly genetic trait, while contributions, above and beyond minP significant loci, are present in ten of thirteen analyses. Further interrogation of these loci, as with any finding from a GWA analysis, is required for confirmation and validation.

**Fig 3:**
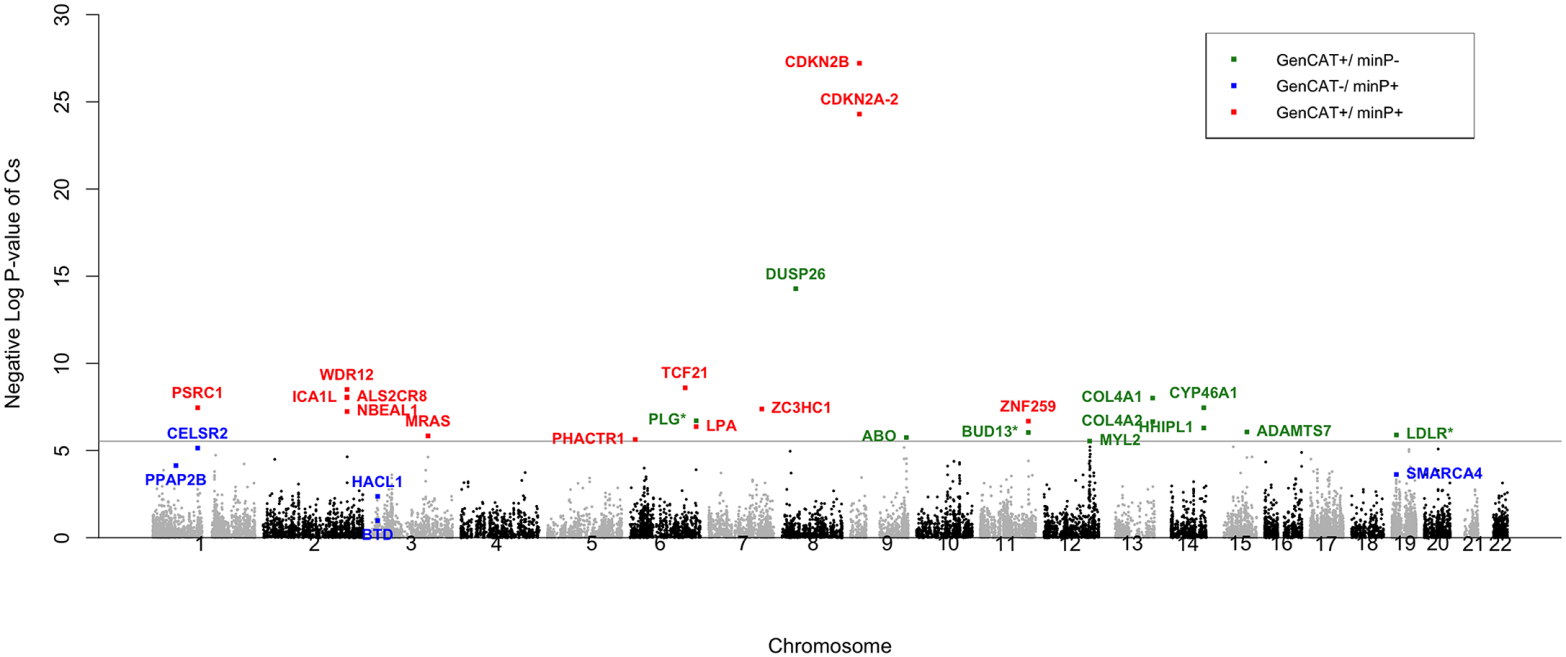
Manhattan plot illustrating GenCAT and minP protein-coding gene discoveries using CARDIoGRAM meta-analysis data. The horizontal line indicates the Bonferroni corrected threshold of 0.05/17280 = 2.89×10^−06^. Genes indicated with * are significant for GenCAT and negative for minP (GenCAT+/minP-) but are within 500Kb of a minP significant gene.

A visual representation of the findings for the CARDIoGRAM analysis is provided in Fig 3. In this Manhattan style plot, the x-axis corresponds to location on the genome and the y-axis is the negative log of the GenCAT p-value. Each dot represents a gene and the horizontal line indicates the adjusted significance threshold. GenCAT positive genes that are minP negative (in green) and denoted with a * are within a 1Mb (±500*Kb*) region of a minP positive gene and are therefore not considered novel GenCAT findings. All of these genes, with the exception of DUP26, are confirmed findings as they are within minP positive regions in subsequent analysis that uses additional data. Additional details on minP significant genes are available in the primary analysis reports [1–3, 7–9].

**Fig 4:**
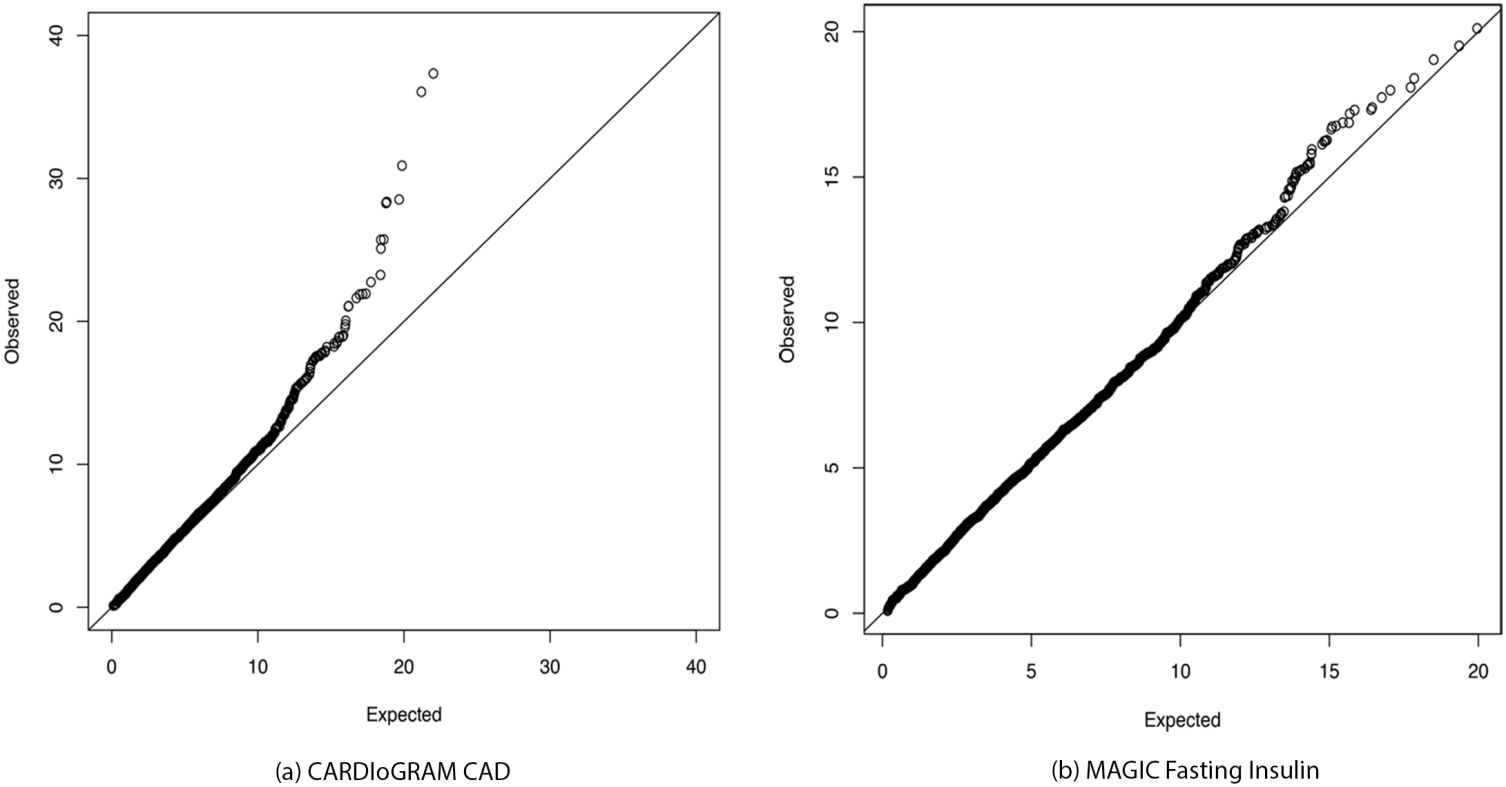
QQ plots of observed versus expected GenCAT test statistics.

Finally, in Fig 4(a) we illustrate the observed GenCAT gene-level test statistics based on CARDIoGRAM CAD as compared to the expected quantiles of a *χ*^2^-distribution. As each statistic has degrees of freedom equal to the number of transformed test statistics, we limit this plot to genes with the median number of 5 statistics after transformation. The expected *χ*^2^-distribution is also based on 5 degrees of freedom. For the purpose comparison, Fig 4(b) illustrates a similar quantile-quantile plot based on the observed GenCAT test statistics based on MAGIC insulin resistance, a trait that resulted in no minP positive or GenCAT positive findings. As expected, the observed test statistics for MAGIC insulin resistance are closer than the CARDIoGRAM CAD statistics to the (*y* = *x*)-line.

#### Pathway analysis using GenCAT

As an additional illustration of the versatility of GenCAT to alternative class-level taxonomies, we applied it to 50 hallmark gene sets in the Molecular Signatures Database (MSigDB) representing well-defined biological processes (http://www.broadinstitute.org/gsea/msigdb/collections.jsp#H) using the CARDIoGRAM data. Here classes are defined as gene sets, and thus we are testing the association of each gene set with the phenotype. For example, we test for association between CAD and the Hallmark Interferon Alpha Response gene set, which is defined as a set of 97 genes (88 genes for which we have available data) that are up-reguated in response to alpha interferon proteins. GenCAT identified 25 of the 50 hallmark gene sets as associated with CAD. The distribution of numbers of genes and SNPs within these sets, as well as *C*_*s*_ and the minimum single SNP p-values, are provided in S6 Table. Although there is likely overlap between gene sets (i.e., the same genes/SNPs will belong to multiple gene sets), we apply a Bonferroni corrected threshold based on the 50 sets considered, which we expect to be conservative. Also included in this table is the strongest gene signal within the corresponding gene set (based on minP) and whether the minimum p-value within this gene is less than a Bonferroni corrected significance threshold based on the number of SNPs (n) in the gene set.

These illustrative pathway analyses are informative in revealing many expected associations, including for example the “bile acid metabolism”, “cholesterol homeostasis”, “coagulation”, “complement” and “DNA repair” gene sets with CAD while suggesting novel relations of CAD with inflammatory (“interferon alpha resp”) and metabolic signaling (“PI3K_AKT_MTOR”) pathways. A broad assortment of sophisticated analytic methods have been described for gene set enrichment analysis (for example, [22–26]) and a formal and comprehensive comparison beyond our illustrative scope here would elucidate the relative advantages and disadvantages of GenCAT in this setting. Here we aim only to illustrate that the analysis of gene sets is one additional application of GenCAT that can be easily and efficiently applied to GWA summary data to further enhance their biological and clinical translation. Further alternative application settings are discussed below.

### Simulation study findings

The estimated FWEC based on 4000 simulations is 0.043 for $C_{s}$, the expected nominal level. The estimated FWEC for minP is 0.016, smaller than the nominal level, which is expected in the context of dependencies because the Bonferroni correction only ensures an upper bound for the FWEC. For comparison, we additionally estimate the FWEC based on a modified QT [12] in which we use Pearson’s correlation between SNPs as the estimated correlation between corresponding test statistics. We note that QT was developed in the context of case control data and in that setting, an alternative estimate of the covariance was applied; however, we expect both estimates to be unbiased. In this case, GenCAT and QT are equivalent with the exception that GenCAT includes a data reduction step as described by Eqs 1 and 2. The FWEC based on 1000 additional simulations, and in this case limiting analysis to testing 100 genes each with less than 20 SNPs for computational purposes, is estimated to be 45.3% for QT and 5.5% for GenCAT using a Bonferonni correction for 100 tests.

The type-1 error rate (based on testing a single gene), on the hand, is close to the nominal level of 0.05 (0.057 for QT and 0.051 for GenCAT). This is a result of the covariance matrix of test statistics being close to singular, and in turn, the tendency of QT to over estimate the gene-level test statistics, resulting in a lack of p-value precision for QT. This instability is addressed with GenCAT through the data projection step which is why GenCAT has appropriate control of the FWEC. We additionally considered the power of QT and GenCAT in this single gene testing setting in which error is appropriately controlled. Notably, in our application of GenCAT we set *ψ* = 0.05, and as described above, QT is equivalent to GenCAT with *ψ* = 0. While the two approaches are comparable for shift parameters of *μ* = 3.0 and greater (power >98% for both approaches), QT consistently performs better than GenCAT for more moderate signals of *μ* = 1 and 2 (empirical power = 64.6% and 96.1%, respectively, for QT and empirical power = 33.7% and 88.2%, respectively, for GenCAT). Importantly, in the GWA multiple testing setting that we are interested in, QT has inflated FWEC as we have shown in simulations, and thus it is not meaningful in this context to compare the power of QT to that of GenCAT.

**Fig 5:**
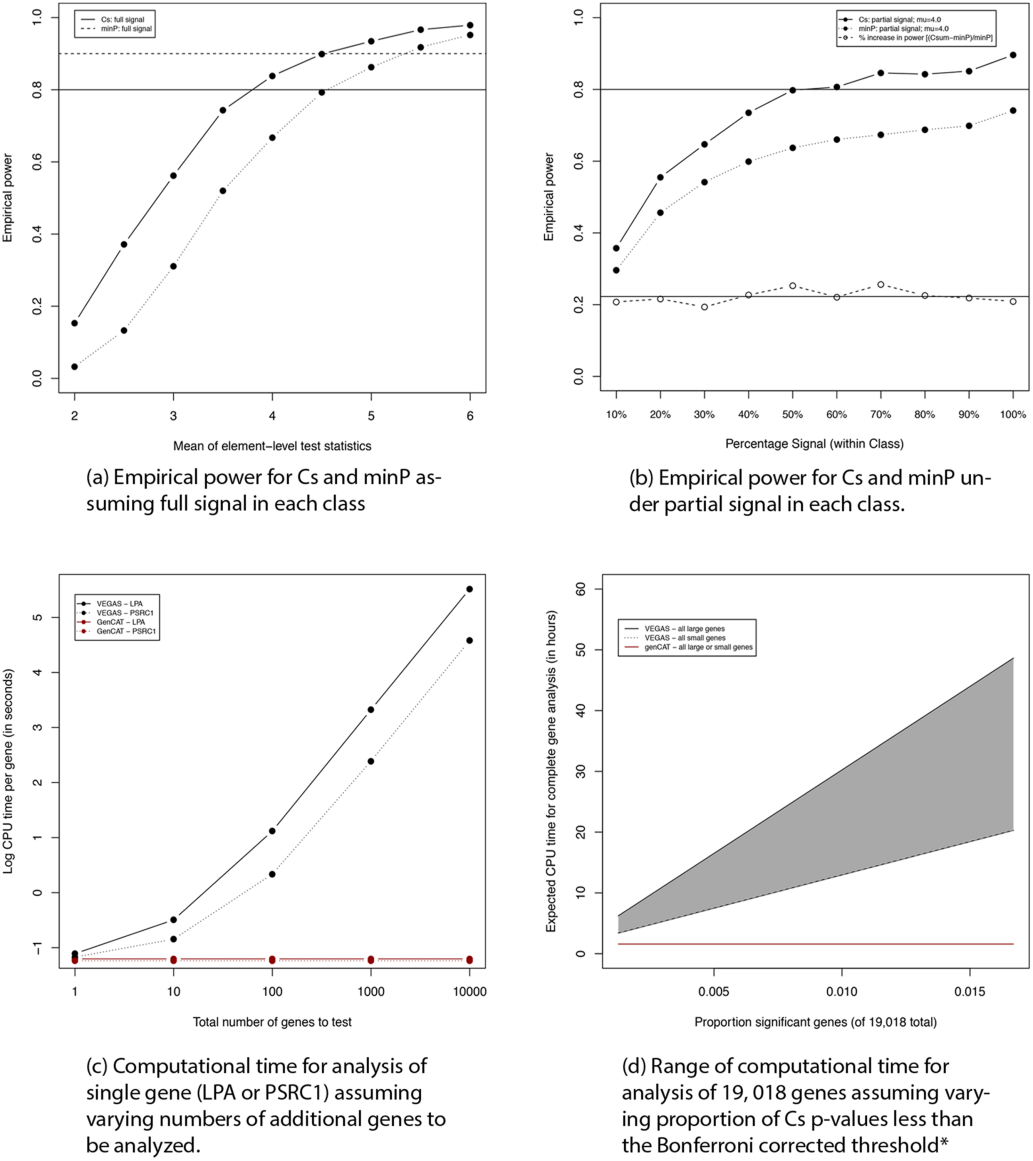
Simulation study results. *We also adjust the p-value distribution to reflect the observed distributions (i.e. estimated proportion of genes for which *p* ≤ 2.6×10^−6^; 2.6×10^−6^ < *p* ≤ 0.001; 0.001 < *p* ≤ 0.1; and 0.1 < *p*) for two complementary data settings: (1) CARDIoGRAM with CAD as the outcome where the estimated proportions are 0.001262, 0.007151, 0.1394 and 0.8521; and (2) GIANT with height, a well-described and highly genetic trait—results not shown, as the outcome where the estimated proportions are 0.01670, 0.02667; 0.1539, and 0.8027. Linear extrapolation is applied to estimate times in between these two extremes in Fig 5(d).

Empirical-based power estimates under a range of conditions are reported and illustrated in Fig 5(a) and 5(b). For computational and practical purposes, we focus the power analysis on genes with less than 100 SNPs and the partial signal analysis additionally excludes genes with only a single SNP. Here we see that if the mean of the element-level test statistics in a class is at least 4.0 and we assume a full signal—i.e. all element-level (single SNP-level) test statistics in the class (gene) arise from a distribution with this mean—then we expect to achieve 80% power to detect the class using the $C_{s}$ statistic (Fig 5(a)). On the other hand, under the same conditions, we achieve 63.5% power with the minP approach (Fig 5(a)). Power of the minP approach approaches the power of $C_{s}$ when *μ* ≥ 6.0. These results are consistent with our expectation that $C_{s}$ would complement minP in the case of moderate signal across multiple elements in a class.

As expected, and illustrated in Fig 5(b), power decreases when we eliminate a percentage of the elements that have a signal, that is, when less than 100% of the element-level test statistics in a class arise from a distribution with non-zero mean. Under partial signal scenarios *C*_*s*_ is consistently about 21% more powerful than minP. While we can not observe the true percentage signal to determine what is most reasonable to assume, our real data analysis results in substantially more findings using the combination of minP and GenCAT than found by minP which is consistent with this observed increase in power under all scenarios for partial signal.

The computational times for implementing GenCAT and VEGAS for a range of conditions are illustrated in Fig 5(c) and 5(d). As shown by the red horizontal line of Fig 5(c), the computational burden associated with the GenCAT analysis of a single gene (whether or not it is statistically meaningful) is not influenced by the total number of genes under study. The computational time associated with VEGAS, on the other hand, for the analysis of a statistically meaningful gene, increases with the total number of genes under investigation. This is a direct result of the VEGAS requirement of more simulations as the Bonferroni corrected threshold for statistical significance is lowered (i.e. when the number of genes under study is increased.) Moreover, this increase in computational burden is greater for larger genes, as illustrated for LPA with 67 SNPs compared to PSRC1 with five SNPs.

In Fig 5(d), we similarly see that the computational time associated with GenCAT analysis of a full set of 19,018 genes is independent of the percentage of significant genes and is relatively constant as the size of the genes under study increases. The computational time for VEGAS, on the other hand, in this setting, increases as the percentage of Bonferroni significant genes increases from 0.0025 to 0.020. Fig 5(d) includes a very approximate range of computational times (shaded region) where the lower bound is based on all genes composed of five SNPs while the upper bound is based on all genes consisting of 67 SNPs. As described in Fig 1, for the three studies we investigate, a plausible range for the number of SNPs in a gene is one to about 5,500 with a mean of approximately 60 and a median of 22−24. The computational times illustrated in Fig 5(d) for both VEGAS and GenCAT are not intended to be highly precise; rather this figure illustrates their relative computational burden under the more extreme scenarios.

Finally, we consider the sensitivity of GenCAT to choice of covariance structure. Herein we focus again on the LPA gene and estimate type-1 error rates and power when data are generated assuming the PennCATH correlation structure for this gene and then analyzed using the GenCAT approach with an estimated correlation structure based on (i) the original PennCATH data; (ii) 1000 Genomes Caucasians (CEU) data; (iii) 1000 Genomes African Americans (ACB) data; (iv) assuming independence. The third and fourth scenarios are clear mispecifications of the covariance matrix while the first scenario is the correct covariance and the second scenario captures sampling variability. Based on 1000 simulations under the null of no association, type-1 error estimates are 0.055, 0.050, 0.075 and 0.222, respectively. Power for detecting shifts of 1, 2 and 3 is estimated to be 48.4%, 99.1% and 100%, respectively, for scenario 1, and slightly lower at 40.8%, 97.9% and 100%, respectively, for scenario 2, and 38.6%, 98.3% and 100%, respectively, for scenario 3. Due to the inflation of type-1-error, the power of scenario 4 is not meaningful and thus not reported.

## Discussion

In this manuscript we presented an approach for analyzing genetic classes that leverages existing data resources, including summary level findings from GWA meta-analysis and knowledge of underlying genetic structure. Our simulations studies suggested that GenCAT can complement the standard post-hoc identification of loci that is based on ascribing genes or loci to statistically significant SNPs. We also demonstrate in our simulation study that use of our theoretically derived test statistic distribution is computationally more efficient than the empirical, simulation based approach that is applied in VEGAS to approximate the theoretical distribution of the test statistic under the null of no association. Given the large number of classes typically under investigation (between 17,280 and 17,406 protein coding genes in the examples provided), precise estimation of the corresponding p-values using empirical distributions requires a large number of simulations, which is computationally burdensome. VEGAS uses a threshold for statistical significance and reports whether each class-level p-value is expected to be above or below the threshold. The GenCAT approach, on the other hand, because it uses a theoretically derived test statistic distribution, allows us to report an accurate quantitative p-value while being computationally efficient. Such a framework also provides a foundation on which to build theoretically rigorous extensions. For example, we are currently investigating modifications to the data transformation step to test gene-environment interactions using statistics corresponding to tests of single SNP-environment interactions in multivariable models.

In addition to investigating protein coding genes, GenCAT can be applied to any of a number of class-level mappings in the pipeline, including for example, epigenetic marks, enhancer elements and non-coding RNAs. Furthermore, SNPs might belong to multiple classes, for example, both a protein coding gene and a non-coding RNA, or both a protein coding gene and an intronic enhancer region. Thus, post-hoc ascribing of statistically significant SNPs to a protein coding gene may be misleading in that the true mechanisms may be via a nested or overlapping non-coding RNA or regulatory element. Indeed, approximately one third of all non-coding RNAs have been identified that overlap with protein coding genes [31, 32]. Analysis of class-level associations thus may better reflect the underlying associations. At the same time, as with any association analysis, translation of any findings requires additional genomic and functional analysis to identify the actual functional element at the locus. A variety of follow-up approaches are typically employed at this stage, including, for example: fine mapping, dense SNP genotyping or DNA sequencing which are used to localize the strongest trait associated SNP in the region; transcriptomics, which examines tissue expression of RNA molecules and facilitates drawing connections between trait associated DNA elements and specific expressed RNAs (e.g. via expression quantitative trait loci or allele specific expression); and functional studies in cell systems and mouse models.

A notable limitation of GenCAT is the relatively low power associated with partial signals in a class, as seen in Fig 5(b). While minP continues to maintain a good portion of its power for detecting classes with at least one very strong signal, it performs relatively poorly in the context of only moderate effects, as shown for *μ* < 4 in Fig 5(a). We are currently developing an extension of GenCAT that allows for testing whether the tail of the test-statistic distribution, e.g. the most extreme 10% of element-level test statistics in a class, is different than we would expect under a null of no association of the class. An additional limitation of GenCAT is that it does not leverage information across all of the classes in making inference about a single class. We described a mixed modeling framework in earlier work [27] that draws strength from the totality of the data to make inference about each gene in a candidate gene setting; however, the relatively small percentage of signal in GWA studies (e.g., 1% of protein coding genes) results in insufficient variability in random class-level effects for straightforward application of this mixed modeling framework to the setting described herein. Finally, apparent false negatives may occur because our current application excludes intergenic regions where approximately half of single SNP signals for some complex traits are found; such intergenic effects can be detected and advanced more efficiently to functional studies through interrogation of specific classes of intergenic regulatory features, such as non-coding RNAs or enhancers. Nonetheless, our investigation suggests that GenCAT may add value to single element association testing when we have moderate signal across the majority of elements in a class. Thus, in summary, we emphasize that GenCAT may serve as a complementary strategy for identifying potentially novel genetic loci, in the forms of specific classes e.g., protein coding genes, non coding RNA, or gene sets or pathways, associated with complex traits.

Our research highlights the importance of routine application of a class-based testing strategy for GWA analysis to complement more standard single-point testing and post-hoc ascribing SNPs to genes and loci. GenCAT is one of an expanding class of analytic methods designed specifically to uncover association in a manner that leverages known structure, e.g. SNP annotations to protein coding genes and the local LD patterns across the genome. We demonstrate that GenCAT is a simple, complementary and efficient strategy for class-level testing that advances existing summary level data resources, and adds significant value with respect to its potential for identifying multiple novel and clinically relevant trait associations.

## Appendix A

GWA studies typically involve fitting a separate linear model for each element *j* (e.g., SNP *j*) under investigation, given in a general form by:

$$\begin{array}{c} y_{i}=\beta_{0}+\beta_{j}x_{ij}+\gamma v_{i}+\epsilon_{i} \end{array}$$

where *i* = 1, …, *m* represents individual, *x*_*ij*_ is commonly coded as 0,1 or 2 for the number of variant alleles observed for the *j*th element in individual *i* and *v*_*i*_ represents some additional clinical or demographic variable for individual *i*. For the ease of presentation, we assume *v*_*i*_ to be a scalar, but it is straightforward to extend the model to the scenario of multiple additional clinical or demographic variables. Also, we assume *ϵ*_*i*_ ∼ *N*(0, *σ*^2^). We can rewrite this model as:

$$\begin{array}{c} y_{i}=\alpha_{0}+\beta_{j}\left(x_{ij}-{\bar{x}}_{j}\right)+\gamma \left(v_{i}-\bar{v}\right)+\epsilon_{i} \end{array}$$

where $\alpha_{0}=\beta_{0}+\beta_{j}{\bar{x}}_{j}+\gamma \bar{v}$. Now let $x_{ij,c}=\left(x_{ij}-{\bar{x}}_{j}\right)$ and $v_{i,c}=\left(v_{i}-\bar{v}\right)$ be centered predictors, and $x_{jc}={\left(x_{1j,c},...,x_{mj,c}\right)}^{T}$ and $v_{c}={\left(v_{1,c},...,v_{m,c}\right)}^{T}$ be the corresponding vectors containing all i=1,⋯,m individuals. Further define $y={\left(y_{1},\cdots ,y_{m}\right)}^{T}$, $1_{m}$ as an *m*-dimensional column of 1’s, *I*_*m*_ as *m* × *m* identity matrix, $D_{c}=(1_{m},v_{c})$ and $M=D_{c}{\left(D_{c}^{T}D_{c}\right)}^{-1}D_{c}^{T}$ to be the orthogonal projection operator onto *D*_*c*_. We then have the estimated regression coefficient for the *j*-th element and its variance as:

$$\begin{array}{c} {\hat{\beta }}_{j}={\left[x_{jc}^{T}\left(I_{m}-M\right)x_{jc}\right]}^{-1}x_{jc}^{T}\left(I_{m}-M\right)y, \end{array}$$

and

$$\begin{array}{c} \text{Var}\left({\hat{\beta }}_{j}\right)={\left[x_{jc}^{T}\left(I_{m}-M\right)x_{jc}\right]}^{-1}\sigma^{2}. \end{array}$$

Thus, given the usually large number of individuals *m* in genome-wide association studies, the test statistic of the significance of ${\hat{\beta }}_{j}$ for the *j*-th element is a *z*-score,

$$z_{j}=\frac{\hat{\beta_{j}}}{\sqrt{Var({\hat{\beta }}_{j})}}=\sigma^{-1}{\left[x_{jc}^{T}\left(I_{m}-M\right)x_{jc}\right]}^{-1/2}x_{jc}^{T}\left(I_{m}-M\right)y.$$

Finally, under the assumption that $x_{jc}⊥v_{c}$, i.e., the *j*-th element *x*_*j*_ is independent of *v*, we have:

$$\begin{array}{c} z_{j}=\sigma^{-1}{\left[x_{jc}^{T}x_{jc}\right]}^{-1/2}x_{jc}^{T}y. \end{array}$$

This assumption, that the covariates are independent of the genotype, is reasonable for the applications we consider. Therefore, for any two elements, *j* and *k*, we can write:

$$\begin{array}{c} \begin{array}{c} \begin{array}{cc} \text{Cov}\left(z_{j},z_{k}\right) & =\sigma^{-2}{\left[x_{jc}^{T}x_{jc}\right]}^{-1/2}x_{jc}^{T}\text{Var}\left(y\right)x_{kc}{\left[x_{kc}^{T}x_{kc}\right]}^{-1/2} \end{array} \end{array}\\ \begin{array}{c} \begin{array}{cc} & =x_{jc}^{T}x_{kc}{\left[\left(x_{jc}^{T}x_{jc}\right)\left(x_{kc}^{T}x_{kc}\right)\right]}^{-1/2} \end{array} \end{array}\\ \begin{array}{c} \begin{array}{cc} & =\frac{\sum_{i=1}^{m}\left(x_{ij}-{\bar{x}}_{j}\right)\left(x_{ik}-{\bar{x}}_{k}\right)}{{\left[\sum_{i=1}^{m}{\left(x_{ij}-{\bar{x}}_{j}\right)}^{2}\sum_{i=1}^{n}{\left(x_{ik}-{\bar{x}}_{k}\right)}^{2}\right]}^{1/2}}=\rho_{x_{j},x_{k}}, \end{array} \end{array} \end{array}$$

where *ρ*_*x*_*j*_, *x*_*k*__ the (*j*, *k*)-th component of sample Pearson’s correlation coefficient matrix, *P*, for elements *j*, *k* ∈ {1, …, *n*} within a class. Thus, we have shown that the variance-covariance matrix of *n* test statistics **z** = (*z*_1_, *z*_2_, …, *z*_*n*_)^*T*^ in Section 3.1, Σ, is given by the sample Pearson’s correlation coefficient *P*.

## Supporting Information

S1 Table Findings of *phase 1—confirmatory analysis*. Locus name is defined arbitrarily based on observed genes in the locus, start and stop are the start and stop coordinates respectively based on Genome Reference Consortium human genome (hg) build 37, n is the number of observed SNPs in the corresponding gene, *K* is the number of SNPs after dimension reduction, Cs is the GenCAT *C*_*s*_ statistic, Cs_p is the corresponding unadjusted p-value, rs_minP is the name of the SNP with the smallest p-value in the corresponding gene, and minP is the minimum observed p-value in the corresponding gene.
(TIF)

S2 Table Summary of GenCAT discoveries in GIANT. Locus name is defined arbitrarily based on observed genes in the locus, start and stop are the start and stop coordinates respectively based on Genome Reference Consortium human genome (hg) build 37, n is the number of observed SNPs in the corresponding gene, *K* is the number of SNPs after dimension reduction, Cs is the GenCAT *C*_*s*_ statistic, Cs_p is the corresponding unadjusted p-value, rs_minP is the name of the SNP with the smallest p-value in the corresponding gene, and minP is the minimum observed p-value in the corresponding gene.
(TIF)

S3 Table Summary of GenCAT discoveries in DIAGRAM—T2D. Locus name is defined arbitrarily based on observed genes in the locus, start and stop are the start and stop coordinates respectively based on Genome Reference Consortium human genome (hg) build 37, n is the number of observed SNPs in the corresponding gene, *K* is the number of SNPs after dimension reduction, Cs is the GenCAT *C*_*s*_ statistic, Cs_p is the corresponding unadjusted p-value, rs_minP is the name of the SNP with the smallest p-value in the corresponding gene, and minP is the minimum observed p-value in the corresponding gene.
(TIF)

S4 Table Summary of GenCAT discoveries in GLGC—2013. Locus name is defined arbitrarily based on observed genes in the locus, start and stop are the start and stop coordinates respectively based on Genome Reference Consortium human genome (hg) build 37, n is the number of observed SNPs in the corresponding gene, *K* is the number of SNPs after dimension reduction, Cs is the GenCAT *C*_*s*_ statistic, Cs_p is the corresponding unadjusted p-value, rs_minP is the name of the SNP with the smallest p-value in the corresponding gene, and minP is the minimum observed p-value in the corresponding gene.
(TIF)

S5 Table Summary of GenCAT discoveries in MAGIC. Locus name is defined arbitrarily based on observed genes in the locus, start and stop are the start and stop coordinates respectively based on Genome Reference Consortium human genome (hg) build 37, n is the number of observed SNPs in the corresponding gene, *K* is the number of SNPs after dimension reduction, Cs is the GenCAT *C*_*s*_ statistic, Cs_p is the corresponding unadjusted p-value, rs_minP is the name of the SNP with the smallest p-value in the corresponding gene, and minP is the minimum observed p-value in the corresponding gene.
(TIF)

S6 Table Summary of GenCAT discoveries of hallmark gene sets in CARDIoGRAM. Twenty-five of 50 hallmark gene sets, defined in the Molecular Signatures Database (MSigDB) (http://www.broadinstitute.org/gsea/msigdb/collections.jsp#H), are detected by GenCAT. *Number of genes in set with at least one available SNP, n is the number of observed SNPs in the corresponding gene set, *K* is the number of SNPs after dimension reduction, Cs is the GenCAT *C*_*s*_ statistic and Cs_p is the corresponding unadjusted p-value. §Strongest gene signal in the gene set is based on minP.
(TIF)

S1 Fig Simulation to estimate correlation of test statistics in GLM setting. A simulation study was conducted to estimate the pairwise correlation between test statistics based on a generalized linear model (GLM) with a logit link. The PennCATH data for 22 observed SNPs in the ADAMTS7 gene were used for illustration. Pairwise Pearson correlations were calculated between each SNP and rs2277547, the SNP with the minimum p-value in ADAMTS7 (shown on the x-axis). For each pair of SNPs, 1400 data points were simulated according to a GLM with a logit link assuming an additive model between each of two SNPs (rs2277547 and one other) and model coefficients of 0.2 and 0.4, respectively. In each case, separate GLMs were then fitted for each SNP to mimic the true analysis approach and corresponding SNP level test statistics were recorded. This simulation was repeated 100 times, each time recording the two SNP level test statistics. The pairwise correlation was then estimated using Pearson’s correlation coefficient. This was repeated for all pairs of SNPs. Finally, the entire procedure was repeated 100 times and the mean correlation and 98% interval (shown on the y-axis) was determined for each pair of SNPs. This is plotted against the original pairwise SNP correlations. This result suggests that, in this setting, Pearson’s correlation coefficient between pairs of SNPs is a reasonable estimate of the correlation between pairs of test statistics. We note that this estimate is commonly used in practice, e.g. [13, 16].
(TIF)
