# Supplementary figures and images for: A Simple Test of Class-Level Genetic Association Can Reveal Novel Cardiometabolic Trait Loci
Source: PLoS One. 2016 Feb 9;11(2):e0148218. doi: 10.1371/journal.pone.0148218 (PMC4747495; doi:10.1371/journal.pone.0148218)

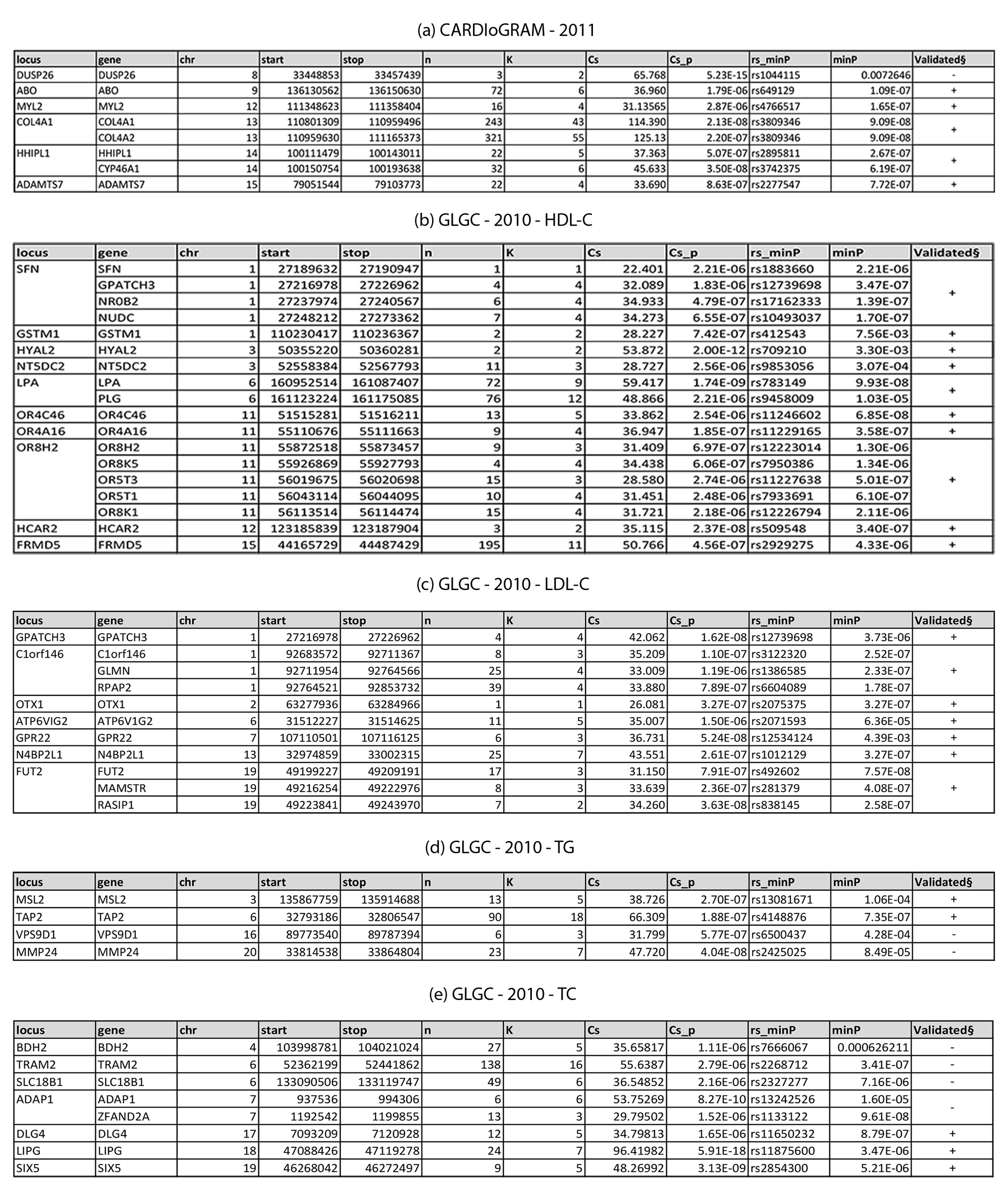

Supplement: S1 Table — Locus name is defined arbitrarily based on observed genes in the locus, start and stop are the start and stop coordinates respectively based on Genome Reference Consortium human genome (hg) build 37, n is the number of observed SNPs in the corresponding gene, K is the number of SNPs after dimension reduction, Cs is the GenCAT Cs statistic, Cs_p is the corresponding unadjusted p-value, rs_minP is the name of the SNP with the smallest p-value in the corresponding gene, and minP is the minimum observed p-value in the corresponding gene. (TIF) [file pone.0148218.s001.tif]

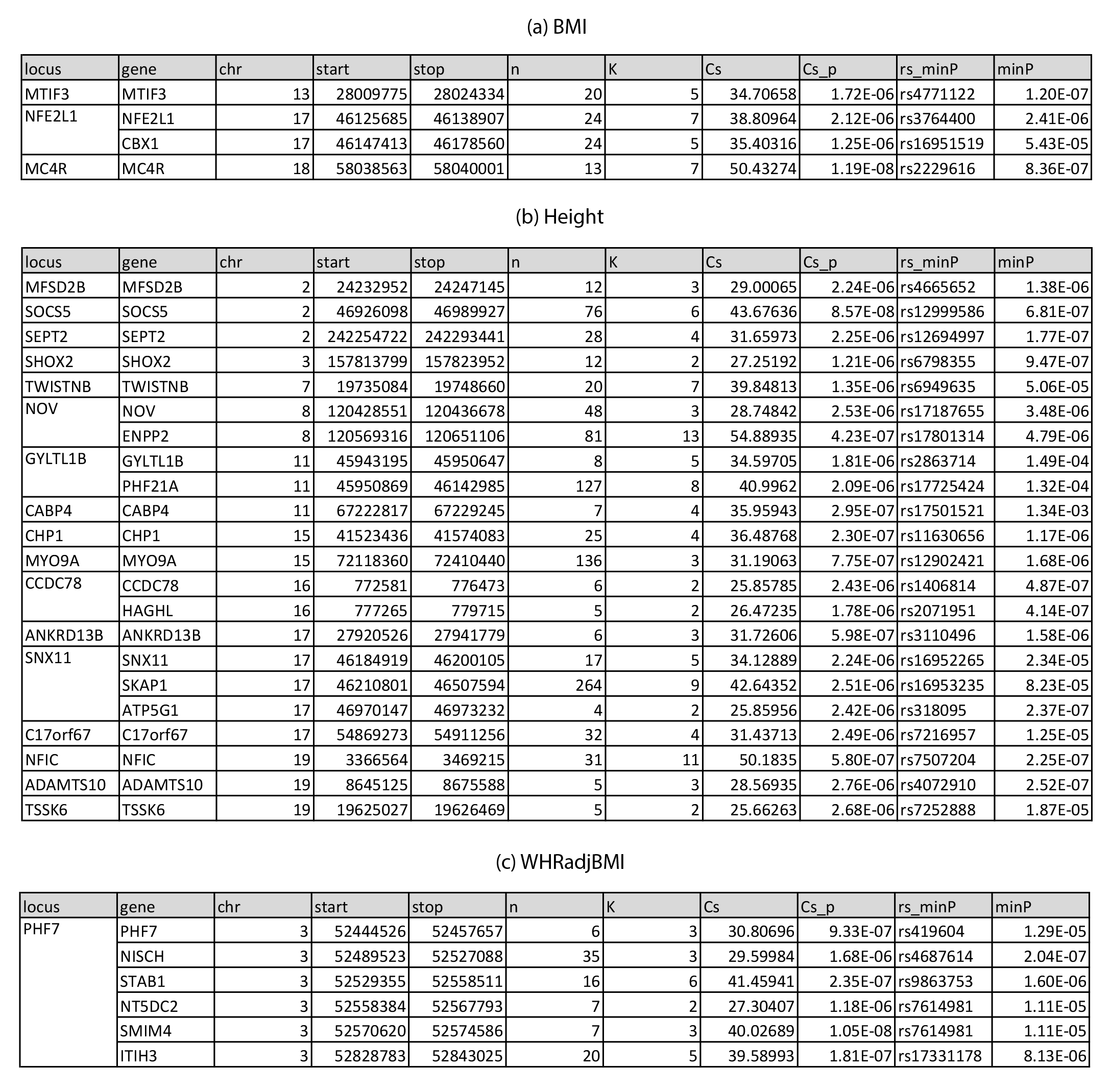

Supplement: S2 Table — Locus name is defined arbitrarily based on observed genes in the locus, start and stop are the start and stop coordinates respectively based on Genome Reference Consortium human genome (hg) build 37, n is the number of observed SNPs in the corresponding gene, K is the number of SNPs after dimension reduction, Cs is the GenCAT Cs statistic, Cs_p is the corresponding unadjusted p-value, rs_minP is the name of the SNP with the smallest p-value in the corresponding gene, and minP is the minimum observed p-value in the corresponding gene. (TIF) [file pone.0148218.s002.tif]

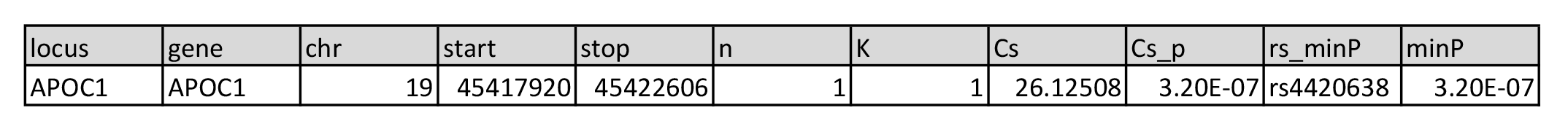

Supplement: S3 Table — Locus name is defined arbitrarily based on observed genes in the locus, start and stop are the start and stop coordinates respectively based on Genome Reference Consortium human genome (hg) build 37, n is the number of observed SNPs in the corresponding gene, K is the number of SNPs after dimension reduction, Cs is the GenCAT Cs statistic, Cs_p is the corresponding unadjusted p-value, rs_minP is the name of the SNP with the smallest p-value in the corresponding gene, and minP is the minimum observed p-value in the corresponding gene. (TIF) [file pone.0148218.s003.tif]

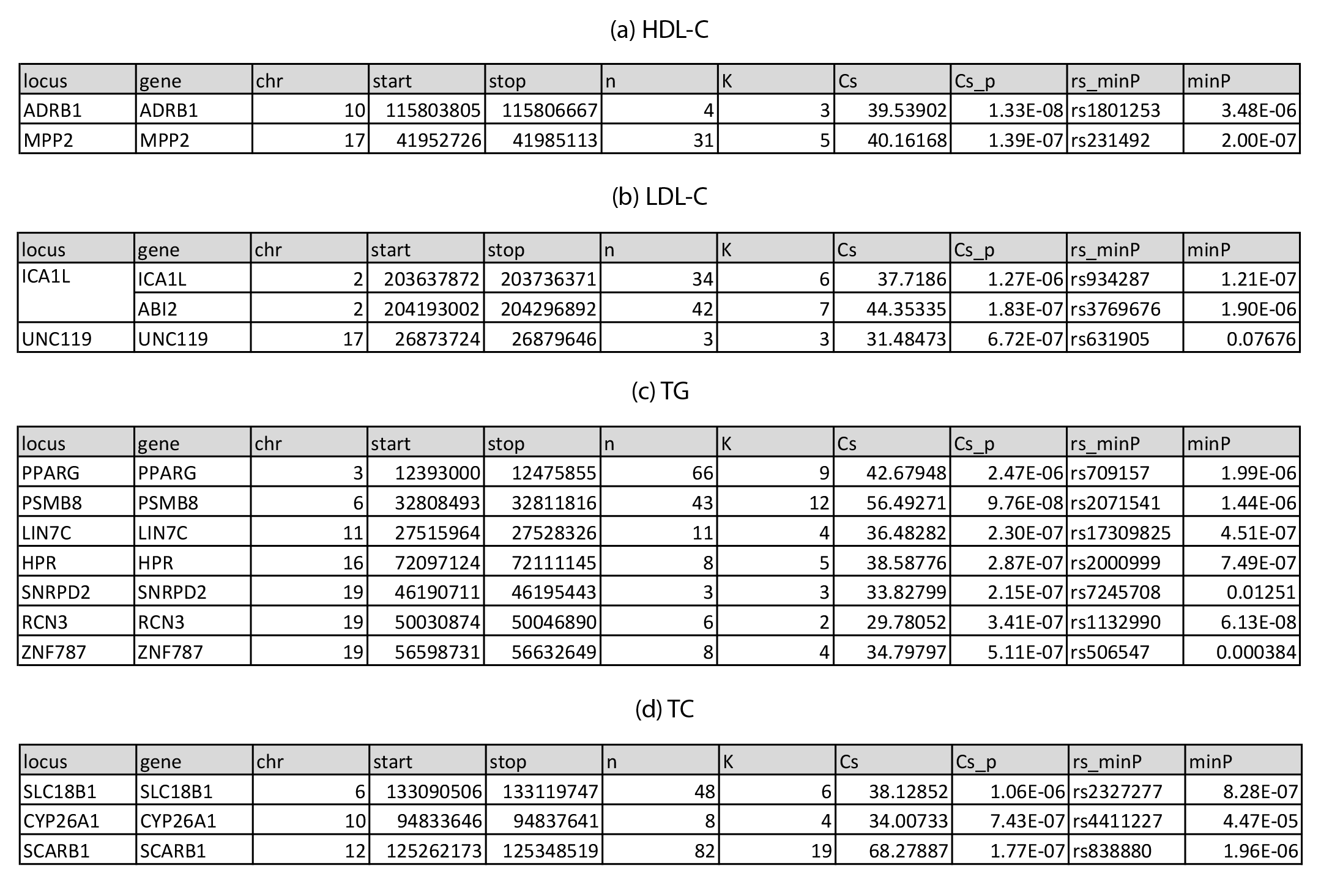

Supplement: S4 Table — Locus name is defined arbitrarily based on observed genes in the locus, start and stop are the start and stop coordinates respectively based on Genome Reference Consortium human genome (hg) build 37, n is the number of observed SNPs in the corresponding gene, K is the number of SNPs after dimension reduction, Cs is the GenCAT Cs statistic, Cs_p is the corresponding unadjusted p-value, rs_minP is the name of the SNP with the smallest p-value in the corresponding gene, and minP is the minimum observed p-value in the corresponding gene. (TIF) [file pone.0148218.s004.tif]

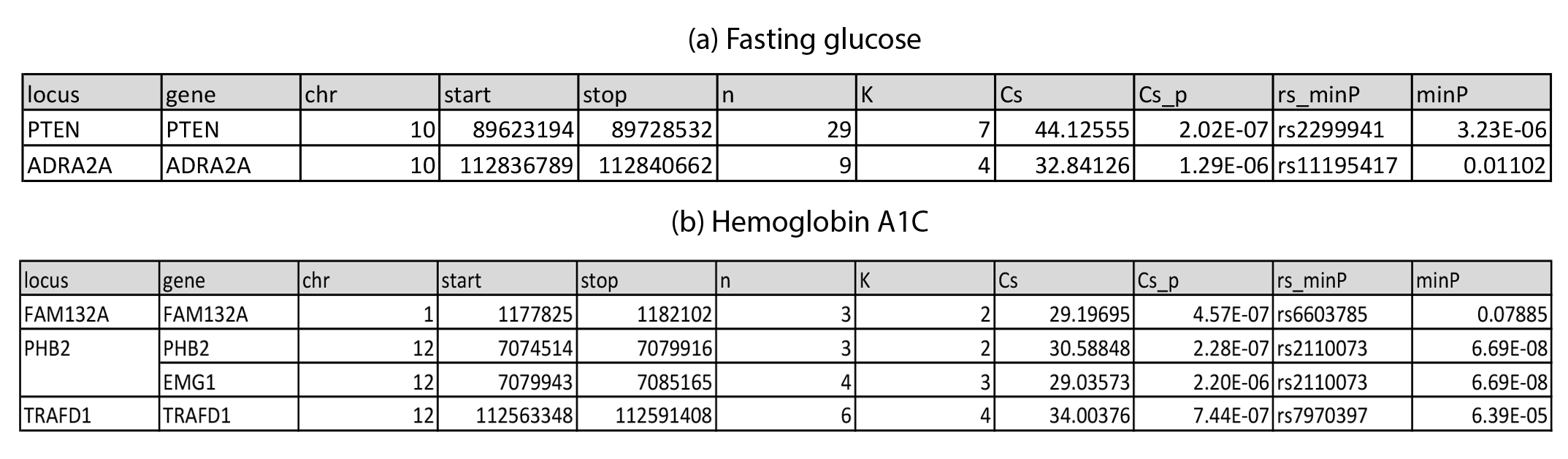

Supplement: S5 Table — Locus name is defined arbitrarily based on observed genes in the locus, start and stop are the start and stop coordinates respectively based on Genome Reference Consortium human genome (hg) build 37, n is the number of observed SNPs in the corresponding gene, K is the number of SNPs after dimension reduction, Cs is the GenCAT Cs statistic, Cs_p is the corresponding unadjusted p-value, rs_minP is the name of the SNP with the smallest p-value in the corresponding gene, and minP is the minimum observed p-value in the corresponding gene. (TIF) [file pone.0148218.s005.tif]

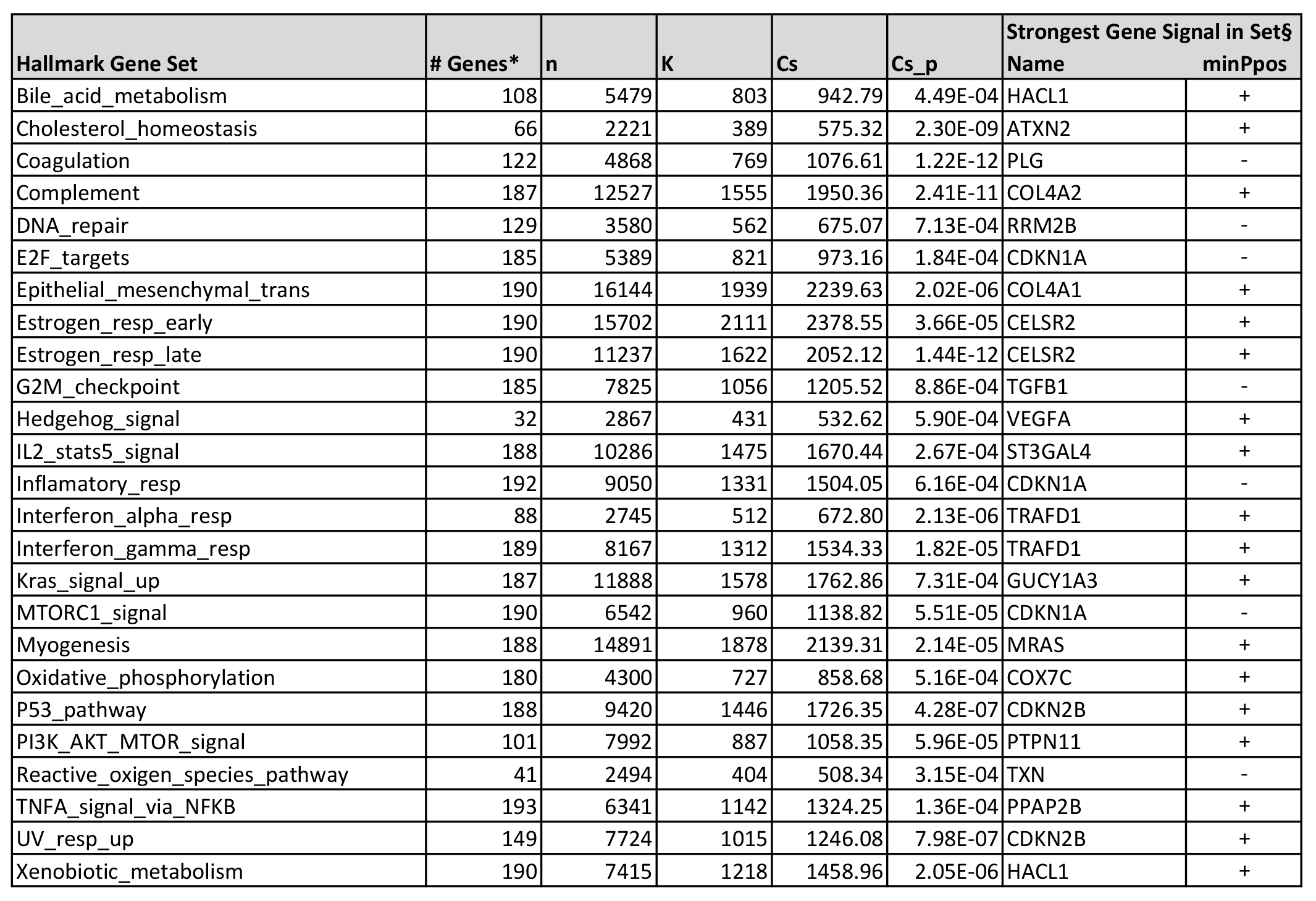

Supplement: S6 Table — Twenty-five of 50 hallmark gene sets, defined in the Molecular Signatures Database (MSigDB) (http://www.broadinstitute.org/gsea/msigdb/collections.jsp#H), are detected by GenCAT. *Number of genes in set with at least one available SNP, n is the number of observed SNPs in the corresponding gene set, K is the number of SNPs after dimension reduction, Cs is the GenCAT Cs statistic and Cs_p is the corresponding unadjusted p-value. §Strongest gene signal in the gene set is based on minP. (TIF) [file pone.0148218.s006.tif]

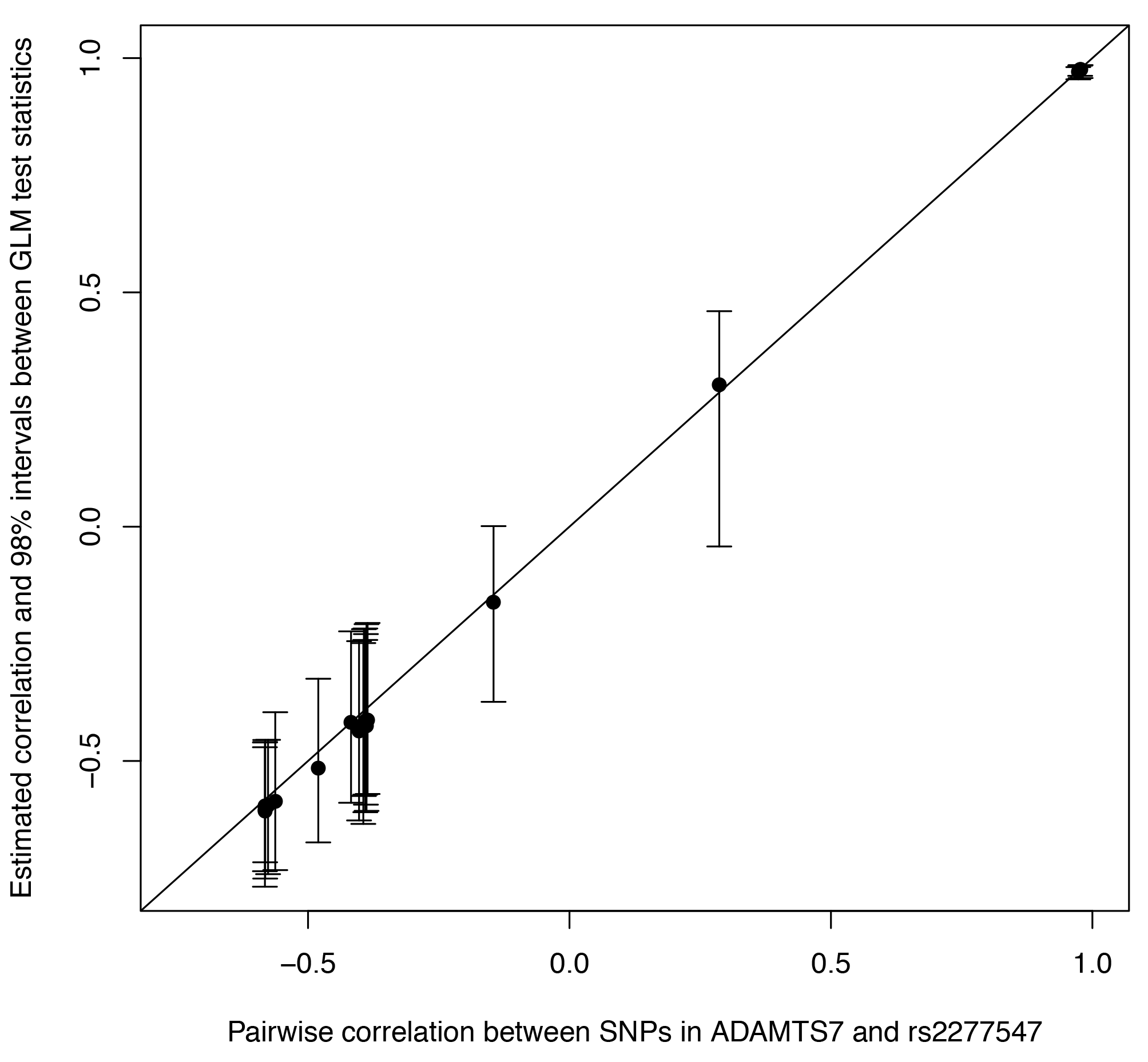

Supplement: S1 Fig — A simulation study was conducted to estimate the pairwise correlation between test statistics based on a generalized linear model (GLM) with a logit link. The PennCATH data for 22 observed SNPs in the ADAMTS7 gene were used for illustration. Pairwise Pearson correlations were calculated between each SNP and rs2277547, the SNP with the minimum p-value in ADAMTS7 (shown on the x-axis). For each pair of SNPs, 1400 data points were simulated according to a GLM with a logit link assuming an additive model between each of two SNPs (rs2277547 and one other) and model coefficients of 0.2 and 0.4, respectively. In each case, separate GLMs were then fitted for each SNP to mimic the true analysis approach and corresponding SNP level test statistics were recorded. This simulation was repeated 100 times, each time recording the two SNP level test statistics. The pairwise correlation was then estimated using Pearson’s correlation coefficient. This was repeated for all pairs of SNPs. Finally, the entire procedure was repeated 100 times and the mean correlation and 98% interval (shown on the y-axis) was determined for each pair of SNPs. This is plotted against the original pairwise SNP correlations. This result suggests that, in this setting, Pearson’s correlation coefficient between pairs of SNPs is a reasonable estimate of the correlation between pairs of test statistics. We note that this estimate is commonly used in practice, e.g. [13, 16]. (TIF) [file pone.0148218.s007.tif]
